# Transcriptomic Identification of *ADH1B* as a Novel Candidate Gene for Obesity and Insulin Resistance in Human Adipose Tissue in Mexican Americans from the Veterans Administration Genetic Epidemiology Study (VAGES)

**DOI:** 10.1371/journal.pone.0119941

**Published:** 2015-04-01

**Authors:** Deidre A. Winnier, Marcel Fourcaudot, Luke Norton, Muhammad A. Abdul-Ghani, Shirley L. Hu, Vidya S. Farook, Dawn K. Coletta, Satish Kumar, Sobha Puppala, Geetha Chittoor, Thomas D. Dyer, Rector Arya, Melanie Carless, Donna M. Lehman, Joanne E. Curran, Douglas T. Cromack, Devjit Tripathy, John Blangero, Ravindranath Duggirala, Harald H. H. Göring, Ralph A. DeFronzo, Christopher P. Jenkinson

**Affiliations:** 1 Division of Diabetes, Department of Medicine, University of Texas Health Science Center at San Antonio, San Antonio, TX, United States of America; 2 Division of Nephrology, Department of Medicine, University of Texas Health Science Center at San Antonio, San Antonio, TX, United States of America; 3 Texas Biomedical Research Institute, San Antonio, TX, United States of America; 4 Division of Orthopedics, Department of Medicine, University of Texas Health Science Center at San Antonio, San Antonio, TX, United States of America; 5 Division of Endocrinology and Diabetes, Department of Pediatrics, University of Texas Health Science Center at San Antonio, San Antonio, TX, United States of America; 6 Division of Clinical Epidemiology, Department of Medicine, University of Texas Health Science Center at San Antonio, San Antonio, TX, United States of America; 7 South Texas Veterans Health Care System, San Antonio, TX, United States of America; 8 School of Life Sciences, Arizona State University, Tempe, AZ, United States of America; University of Padova, ITALY

## Abstract

Type 2 diabetes (T2D) is a complex metabolic disease that is more prevalent in ethnic groups such as Mexican Americans, and is strongly associated with the risk factors obesity and insulin resistance. The goal of this study was to perform whole genome gene expression profiling in adipose tissue to detect common patterns of gene regulation associated with obesity and insulin resistance. We used phenotypic and genotypic data from 308 Mexican American participants from the Veterans Administration Genetic Epidemiology Study (VAGES). Basal fasting RNA was extracted from adipose tissue biopsies from a subset of 75 unrelated individuals, and gene expression data generated on the Illumina BeadArray platform. The number of gene probes with significant expression above baseline was approximately 31,000. We performed multiple regression analysis of all probes with 15 metabolic traits. Adipose tissue had 3,012 genes significantly associated with the traits of interest (false discovery rate, FDR ≤ 0.05). The significance of gene expression changes was used to select 52 genes with significant (FDR ≤ 10^-4^) gene expression changes across multiple traits. Gene sets/Pathways analysis identified one gene, alcohol dehydrogenase 1B (*ADH1B*) that was significantly enriched (P < 10^-60^) as a prime candidate for involvement in multiple relevant metabolic pathways. Illumina BeadChip derived *ADH1B* expression data was consistent with quantitative real time PCR data. We observed significant inverse correlations with waist circumference (2.8 x 10^-9^), BMI (5.4 x 10^-6^), and fasting plasma insulin (P < 0.001). These findings are consistent with a central role for *ADH1B* in obesity and insulin resistance and provide evidence for a novel genetic regulatory mechanism for human metabolic diseases related to these traits.

## Introduction

The global twin pandemics of obesity and type 2 diabetes (T2D) represent a major social, economic, medical, and research challenge through the current century. Approximately 26 million people in the United States (US) are estimated to have diabetes, and about 48 million people are projected to have diabetes by the year 2050 if current demographic trends persist [1]. In 2010, 79 million US adults 20 years or older were estimated to have prediabetes (26% of the population) [1] and 36% of US adults were obese [2]. The prevalence rates of T2D and obesity are particularly high in US ethnic minorities such as the Mexican American population [2]. T2D is a complex metabolic disease, characterized by insulin resistance (IR) and impaired β-cell function [3–5]. In its early “pre-diabetes” stage, elevated glucose levels co-occur with elevated insulin due to defective insulin responses in insulin target tissues, notably skeletal muscle, liver and fat, and by defects in insulin secretion from pancreatic β-cells [4, 5]. We previously showed that Mexican Americans have a high genetic predisposition for IR, T2D and related conditions [6, 7]. We also have shown that compensatory hyperinsulinemia is an early metabolic change that precedes the onset of hyperglycemia and overt T2D, and represents a physiological response offsetting IR. This compensatory hyperinsulinemia manifests as an increase in fasting plasma insulin (FPI) in normoglycemic subjects with a positive family history of T2D [8, 9]. In typical T2D, individuals pass through a pre-diabetes “gate”, characterized by IR, increased FPI, and elevated glucose, prior to the development of overt T2D, which is eventually accompanied by a progressive decline in insulin secretion following Starling’s Curve of the pancreas, originally described by DeFronzo et al. [10]. IR is an underlying factor that co-occurs with a cluster of highly correlated traits including obesity, T2D, hypertension (HTN), and dyslipidemia (DL). This cluster of traits is referred to as the insulin resistance, or metabolic, syndrome (MS) and is a predictor of cardiovascular disease and stroke. One pervasive form of insulin resistance, obesity, is a major risk factor for T2D [10, 11].

Whole body IR includes a range of tissue-specific metabolic abnormalities which are linked by a common failure to respond to insulin. Primary tissues involved are skeletal muscle, liver, adipose tissue, and pancreatic β-cells, and these are augmented by the gut and brain. The two key endocrine tissues involved are the pancreas and adipose tissue. Pre-diabetes is an insulin resistant state that typically precedes diabetes and may lead to T2D when accompanied by a primary defect in the pancreatic β-cells.

Both genetic and environmental factors play important roles in the development of T2D [3, 12–15]. There have been continuing efforts to localize and characterize T2D susceptibility genes using a variety of approaches: genome-wide linkage (GWL), genome-wide association studies (GWAS), whole genome sequencing (WGS) and genome-wide gene expression (transcriptomics). Transcriptomic studies provide a strategy for moving from gene localization towards direct gene characterization and functional analysis. The BeadArrays used in the present study included oligonucleotide probes for a total of 47,324 transcripts, which cover most known genes plus several common splice variants and small non-coding RNAs [16, 17].

In recent years the genome-wide association study (GWAS) has become a popular study design with several notable successes in localizing novel putative T2D susceptibility genes/variants [18]. This approach is based on the common variant/common disease hypothesis, and generally uses data from samples of unrelated cases and controls rather than from related family members. So far, more than 70 [19] T2D susceptibility genes have been localized with genome-wide significance [20–23]. Of these, the most significant gene was the transcription factor 7-like 2 (*TCF7L2*) which was localized in a follow-up study to our previous report of linkage of T2D to the 10q chromosomal region in Mexican Americans [24–26]. However, in most cases the identity of the causal genes and the functional relevance of the implicated genetic variants have yet to be established. As has been the case with various GWASs of other complex diseases, the genetic contribution of common variants identified by the GWASs to the overall susceptibility to T2D is rather modest. Most T2D GWASs to date have involved populations of European ancestry and their relevance to other ethnic groups remains uncertain. Mexican Americans, who are at high risk of T2D, have been examined to a limited extent to resolve issues such as allele frequency and linkage disequilibrium differences among diverse populations [27].

We previously demonstrated modulation of the expression of IR-associated inflammation genes in skeletal muscle from normal glucose tolerant (NGT) subjects in response to acute hyperinsulinemia during an insulin clamp.[28] We also reported a marked fibrotic inflammatory response in skeletal muscle in response to lipid-induced IR.[29] Using DNA microarrays we observed increased expression of extracellular matrix genes, and increases in the corresponding proteins, following a 48 hour lipid infusion[30] and described common alterations in expression of genes in the first global comparison of the skeletal muscle transcriptome and proteome[31] to evaluate the relationship between RNA and protein expression in the pathophysiology of IR. These studies provided evidence that transcriptomic data can provide valuable information to generate novel hypotheses regarding muscle function in disease states including insulin resistance. Those findings were extended to adipose tissue in the present study.

Fat is one of the largest tissues in the body with a normal mean of 18–24% body weight in males, 25–31% in females and >30% in obese individuals. It is essential for normal energy homeostasis. Dysregulation of normally insulin-sensitive adipose tissue function has been repeatedly shown to be involved in whole body IR, lipotoxicity, obesity and T2D [32, 33]. In addition to its role in energy storage and mobilization in response to fluctuating energy demands, adipose is a dynamic endocrine tissue producing a variety of potent adipokines which interact with other tissues, including skeletal muscle, liver, brain and pancreatic β-cells. Under energy sufficient states insulin promotes the uptake of glucose in fat, its conversion to triacylglycerol for storage, and a decrease in lipolysis of triacylglycerols to produce circulating free fatty acids and glycerol. In energy deficient states, under the control of insulin-antagonistic counterregulatory hormones, including glucagon, these processes are normally reversed such that energy is released from fat stores as free fatty acids for β-oxidation and energy production in skeletal muscle and liver. The term “adiposopathy” or “sick fat” has been used to describe a state of metabolic dysregulation wherein fat becomes resistant to the effects of insulin, failing to take up glucose and switch off lipolysis in response to insulin and producing inappropriate amounts of adipokines [32]. Adipocyte IR is a complex metabolic disease leading to excess accumulation of triacylglycerols in multiple fat depots, and dysregulated hormonal interaction with other tissues. Thus, adipose IR contributes to whole body IR, obesity and T2D [33].

To date there have been no reported systematic studies of adipose tissue genome-wide gene expression (transcriptome) and correlation with clinical metabolic traits in Mexican Americans. Therefore, the purpose of this study was to perform genome-wide transcriptomic measurements, to define T2D susceptibility genes and their potential functions in Mexican Americans from the Veterans Administration Genetic Epidemiology Study (VAGES) residing in San Antonio, Texas.

## Results

In this study, we first screened the genome for expression of more than 47,000 genes and transcripts, following our previous findings of strong genetic correlations underlying T2D, obesity and IR traits in Mexican Americans from the VAGES (**S1 and S2 Tables**). We screened the transcriptomes of 308 individuals recruited from the VAGES (**Table 1**) in three tissues: (fat, skeletal muscle and leukocytes). The results presented here were based on transcriptomic data from abdominal subcutaneous adipose tissue biopsies from 75 individuals.

**Table 1:** Characteristics of VAGES Gene Expression Study Participants for Selected Metabolic Syndrome (MS)-Related Traits.

Variable	N	Mean ± SD or %
Females	308	66.6
Age (years)	308	49.4 ± 12.7
BMI (kg/m^2^)	307	34.0 ± 7.7
WC (mm)	305	105.6 ± 18.4
T2D	305	41.3
Pre-T2D [ND]	179	69.3
FPG (mg/dl)	305	141.7 ± 63.5
HbA1c	301	6.6 ± 1.8
FPI [ND] (μIU/ml)	176	8.4 ± 6.7
HOMA-IR [ND]	176	1.1 ± 0.9
Matsuda ISI	293	5.2 ± 6.5
Adipo-IR	294	70.4 ± 62.2
TC (mg/dl)	302	186.7 ± 39.6
HDL (mg/dl)	302	46.1 ± 15.5
TG (mg/dl)	302	143.6 ± 122.2
SBP (mm Hg)	306	133.0± 17.1
DBP (mm Hg)	305	73.5 ± 10.4

ND = Non diabetics; BMI = body mass index; WC = waist circumference; FPG = fasting plasma glucose; HbA1c = glycated hemoglobin; FPI = fasting plasma insulin; HOMA-IR = homeostasis model assessment of insulin resistance; Matsuda-ISI = Matsuda insulin sensitivity index; Adipo-IR = adipocyte insulin resistance index; TC = total cholesterol; HDL = high density lipoprotein cholesterol; TG = triglycerides; SBP = systolic blood pressure; DBP = diastolic blood pressure.

### RNA Isolation and Generation of Gene Expression Data

We recalled 308 Mexican American participants from the VAGES for this study. Their clinical and anthropometric characteristics are shown in **Table 1**. The gene expression dataset was representative of the entire VAGES cohort except that the proportion of individuals with T2D was lower because we selected mainly non-diabetic participants for the transcriptomic/physiologic studies. Abdominal subcutaneous adipose tissue (FAT) biopsies were obtained from a subset of 75 unrelated non-diabetic individuals. In addition, we obtained whole blood (WBCs) samples from 308 individuals and skeletal muscle (SKM) biopsies from 74 non-diabetic individuals. All tissue samples were obtained under fasting conditions in the morning during the participant’s clinic visit. RNA samples were used for preparation of cDNAs, and expression of transcripts was measured using Illumina BeadArrays (Human HT-12 V4) in three batches (275, 39, and 168 samples, respectively, with each batch containing samples from all 3 tissues). All but one FAT sample provided high quality gene expression data. Significantly expressed probes were identified within each tissue and batch at a false discovery rate (FDR) set at 5%, and significant probes in any tissue and batch were kept for full processing, so that the probe set includes all relevant probes and the probe sets are identical between tissues for ease of comparison. Subsequently, the expression values for these probes were subjected to background noise subtraction, log_2_ transformation, and three rounds of quantile normalization (within tissue of a given batch, across tissues in a given batch, and across batches). A total of 31,545 probes (67% of all probes) were significantly expressed in at least one tissue and batch. Gene expression data were processed and normalized as described in the Material and Methods section.

### Identification of Probes and Genes Whose Expression was Significantly Correlated with Clinical Traits

**Fig 1:**
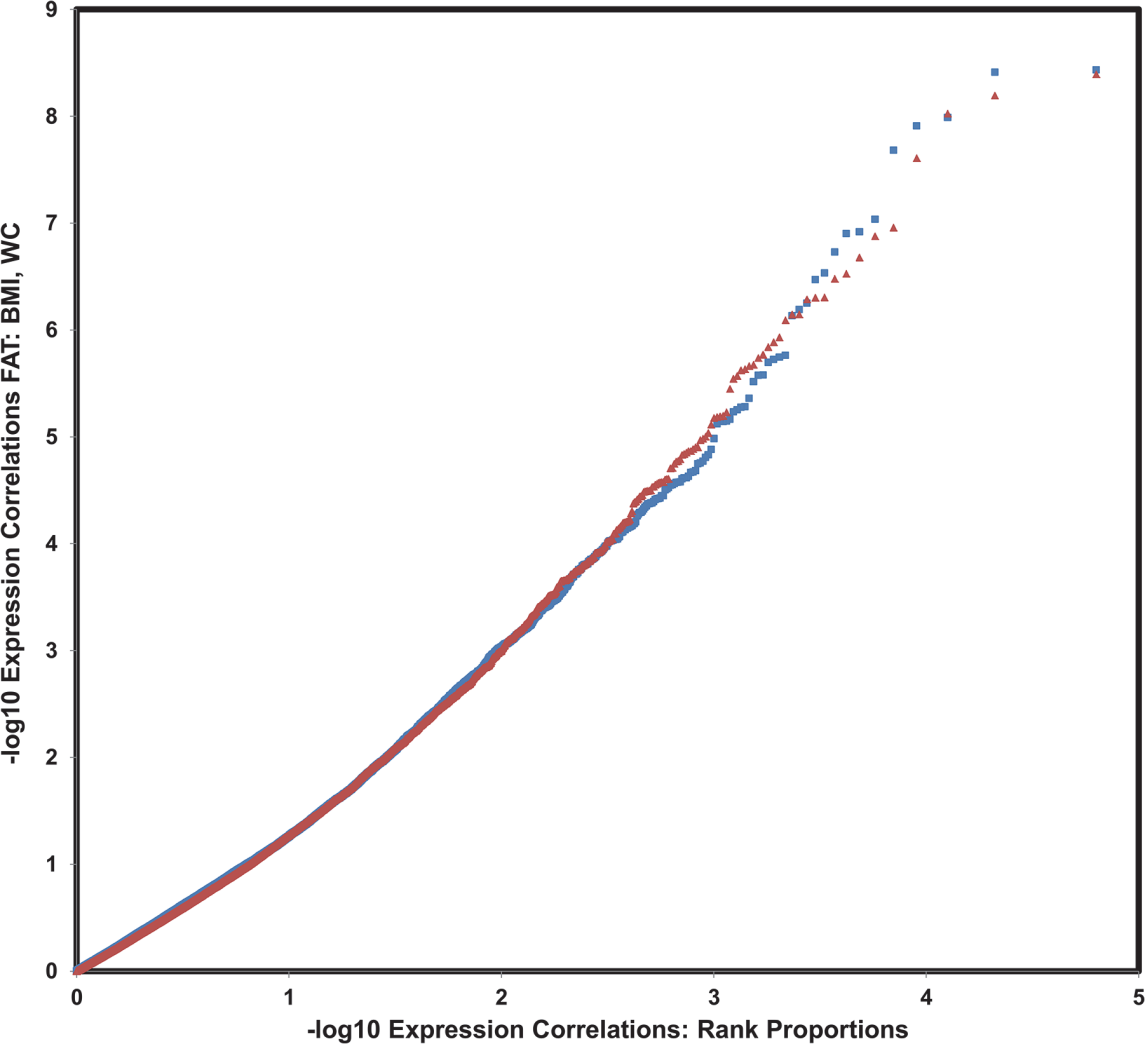
Quantile-Quantile (QQ) plots of Observed vs Expected P-Values of Regression Analysis between Probe Expression Levels and BMI and WC in FAT Tissue. BMI is shown in blue) and WC in red. Expected p-values on the x axis are calculated as the minus log_10_ rank proportion. A total of 35,541 probes are shown. The most significantly correlated genes are indicated by marked deviations from the diagonal in the upper rate.

After full processing, we conducted linear regression analysis on each probe’s expression in FAT separately, in order to identify those probes whose expression levels were significantly correlated with 15 clinical traits: preT2D, HbA1c, HOMA-IR [34], the Matsuda insulin sensitivity index (ISI) [35], FPI, FPG, BMI, TG, WC, HDL, LDL, TC, FFA, SBP and DBP**.** Sex, age, and two batch indicator variables were included as additional variables in the multiple regression model. For ease of summary and interpretation, all probe names were converted to gene names and symbols using the “Convert” function of Gene Profiler[36], and the results were curated by hand to remove all potential double gene entries and other errors; genes with multiple probes were counted only once. **Table 2**shows the number of significant probes at a FDR 0.05 for each of the examined traits. Eight of fifteen traits had significant gene expression findings, with the highest number of significant probes (1,437) being observed for FPI. **Fig. 1**shows a quantile-quantile (QQ) plot of observed vs. expected p-values for BMI (blue) and WC (red) in FAT tissue. Note that the curves follow the null distribution at first, but then exhibit an upward curvature. The dots on the upper right correspond to the many FDR ≤ 0.05 significant hits (175 and 154 for these two traits, respectively). These significant hits appear to be only the tip of the iceberg, given the fairly early deviation of these curves from the diagonal, which indicates an elevated rate of small p-values compared to the null expectation

**Table 2:** Counts of Probes with Significant Gene Expression Differences by Examined Traits.

Trait	P	N (FDR ≤ 0.05)
**BMI**	1.1x10^-8^	175
**WC**	7.5x10^-9^	154
**SBP**	5.7x10^-5^	0
**DBP**	1.1x10^-4^	0
**Chol**	1.4x10^-4^	0
**HDL**	1.6x10^-7^	85
**TG**	2.0x10^-5^	0
**FFA**	3.6x10^-5^	0
**preT2D**	6.8x10^-6^	0
**HbA1c**	5.3x10^-10^	35
**ISI**	1.2x10^-7^	33
**HOMAIR**	4.2x10^-9^	1,058
**FPG**	3.8x10^-9^	34
**FPI**	2.4 x 10^-8^	1,437

Traits are shown in the first column. Gene counts for False Discovery Rate (FDR) ≤ 0.05 are shown in column 3. Multiple regression analysis was conducted on individual probes, while including age, sex, and batch number (2 indicator variables) as variables in the model.

### Overlap in FAT Gene Sets between Pairs of Traits

**Table 3**shows a tabulation of the number of significantly (FDR ≤ 0.05) expressed genes (lower triangle) between pairs of traits. The proportions of significantly expressed genes shared in common (overlapping) between each pair of traits is shown in the upper triangle. Each proportion was calculated with respect to the smaller of the two sets of genes in each trait pair. The largest proportion of genes shared in common was between FPI and HOMA-IR (94%, 992 genes) followed by FPG and HbA1c (85%, 29 genes), and BMI and WC (69%, 106 genes). The large numbers of significantly expressed genes in common between these trait pairs was not surprising given the known physiological relationships between these pairs of traits. For example FPI is a marker of IR and a factor used in the calculation of HOMA-IR; FPG and HbA1c represent levels of blood glucose and glycated hemoglobin (which is dependent on levels of blood glucose); and BMI and WC represent related measures of obesity. HbA1c- and FPG-related genes did not overlap with any other traits. Both FPI and HOMA-IR also overlapped with BMI, WC, HDL and ISI, with 21–50% of genes shared in common within each trait pair. Both BMI and WC overlapped significantly with HDL and ISI, with overlaps of 18–26%. Based on the overall proportion of significantly expressed genes in common, the most strongly related traits were clearly HOMA-IR/FPI/WC/BMI in one “IR cluster” and HbA1c/FPG in a second “glycemic cluster”. In summary, IR-related traits were strongly related by significant differential expression of genes in common.

**Table 3:** Overlap among Genes with Significant Expression Differences by Pairs of Traits.

Trait	FPI	HOMA	FPG	HbA1c	BMI	WC	HDL	ISI	
**FPI**	**1,437**	0.94	0.00	0.03	0.45	0.50	0.27	0.27	
**HOMA**	992	**1,058**	0.00	0.03	0.35	0.40	0.21	0.24	**Proportion**
**FPG**	0	0	**34**	0.85	0.00	0.00	0.00	0.00	**of genes**
**HbA1c**	1	1	29	**35**	0.00	0.00	0.00	0.00	**per**
**BMI**	79	61	0	0	**175**	0.69	0.26	0.18	**trait**
**WC**	77	62	0	0	106	**154**	0.26	0.18	**pair**
**HDL**	23	18	0	0	22	22	**85**	0.03	**overlap**
**ISI**	9	8	0	0	6	6	1	**33**	
		**Gene counts per trait pair overlap**							

The first row and column show the traits examined. The diagonal from left to right shows the number of significantly differentially expressed genes per trait, in bold, taken from Table 2.The numbers of significantly (FDR ≤ 0.05) expressed genes in common between each trait pair are shown below the diagonal and the proportions of significantly expressed genes which overlap between each trait pair are shown above the diagonal. Proportions were calculated relative to the smaller of either of the two sets of genes in each trait pair.

### Selection of Genes for Gene Sets / Pathways Analysis

To obtain a list of the most significant genes from adipose tissue for deeper analysis, we used an FDR cutoff of 10^–4^, which produced a set of 52 significant “gene”-trait pairs, ranging in significance from FDR values of 10^–4^–10^–7^. These genes were spread across a total of five traits, with some of the genes being significantly correlated with two or three related traits. When the duplicate and triplicate genes were taken into account, there remained 36 unique probes. Probe IDs were converted to gene names manually using the Illumina v4 probe list. Several genes were interrogated by more than one unique probe (4 genes had 2 probes each and one gene had 3 probes) and there was one probe with no current annotation (removed from Ensembl in 2009). When these were accounted for, the list of remaining unique genes was 29 (i.e. 36 probes—[6 multiplicate probes + 1 non-functional probe] = 29 final genes) (**Table 4**). Only one gene, *ADH1A*, was shared in common between 3 traits (BMI, WC and FPI). Eleven genes were shared in common with two traits: six with HbA1c plus FPG; four with BMI plus WC; and one with WC plus FPI. The genes with significant expression across more than one trait were of particular interest since they could potentially underpin general mechanisms associated with IR / obesity in adipose tissue.

**Table 4:** Candidate Gene List.

Gene	Minimum FDR across Traits	Significantly Correlated Traits
MYL1	3.45E-06	HbA1c, FPG
MYL2	5.76E-06	HbA1c, FPG
KLHL41	5.76E-06	HbA1c, FPG
ADH1B	9.97E-05	BMI, WC
FRZB	9.97E-05	BMI, WC
MYBPC1	9.51E-05	FPG
AZGP1	9.97E-05	BMI, WC
NMNAT2	9.70E-05	WC
ADH1A	6.97E-04	FPI, WC, BMI
GPD1L	1.95E-04	BMI, WC
CABC1	2.41E-04	FPI, WC
NEB	4.52E-04	HbA1c, FPG
MB	4.52E-04	HbA1c, FPG
TPM2	6.56E-04	HbA1c, FPG
ANGPT2	1.31E-04	WC
MYH7	1.61E-04	HbA1c
AKAP1	2.41E-04	FPI
NRIP3	2.41E-04	FPI
TNNC2	3.36E-04	HbA1c
NEK6	3.52E-04	FPI
QPRT	3.52E-04	FPI
MYH2	4.08E-04	FPG
CKM	4.52E-04	HbA1c
BMS1	4.83E-04	WC
SNX3	5.14E-04	FPG
HADH	6.54E-04	WC
ENO3	8.64E-04	HbA1c
PXMP2	9.26E-04	WC
NTRK3	9.47E-04	BMI

Shown are the genes whose expression level (based on one or more probes) was significantly correlated with one or more clinical traits at FDR ≤ E-04. Genes (N = 29), indicated by their official HGNC symbol in the first column, are ordered from top to bottom according to the FDR value in the second column and secondly by the number of shared traits shown in the third column.

### Gene Sets / Pathways Analysis

The final list of 29 genes from adipose tissue was analyzed using the Gene Group Functional Profiling tool in Gene Profiler to search for significant enrichment of gene sets within Gene Ontology (GO) categories, including gene function, subcellular location, intracellular pathways, interaction of gene products with other proteins, associated transcription factors based on high-throughput data analysis of information from multiple large, gene / RNA / protein / interaction databases. Purely *in silico* annotation was excluded from analysis to minimize potentially spurious results. Following this analysis, two closely related genes, *ADH1A* and *ADH1B* (see below), were significantly enriched in GO categories potentially related to IR/obesity (**Table 5**). They were the only genes to be significantly associated with putative IR/obesity related mechanisms and pathways and fulfilled multiple related GO criteria for potential involvement in glycolysis and FFA metabolism. No other genes met these criteria. Several other genes scored highly on mechanisms related to cellular structure and muscle contraction. While these potential housekeeping genes cannot be completely excluded from consideration, the likelihood of their involvement in IR/obesity metabolism is smaller. We repeated these gene sets / pathway analyses using the WEB-based Gene Set AnaLysis Toolkit (WebGestalt) analytical engine (http://bioinfo.vanderbilt.edu/webgestalt/) and the Kyoto Encyclopedia of Genes and Genomes (KEGG) database (www.genome.jp/kegg, accessed 8 December, 2013). Despite the use of this alternative statistical test for enrichment, the results were essentially identical (results not shown). For completeness we next analyzed the gene sets significant at FDR ≤ 0.05 for the 8 traits with any significant genes (see Table 2) containing from 33–1437 probes (data not shown). We found little to no improvement in significance for the key GO terms related to glycolysis and FFA metabolism despite, in several cases, an increase in the number of probes within these specific categories. For example, for 175 BMI genes, the number of genes for fatty acid and glycolysis metabolism increased from two to five (with 3 *different* additional genes in each case) with *P* values of 3.81 x 10^–3^ and 4.25 x 10^–2^, respectively, representing no improvement in significance for pathway enrichment. This increased our confidence that the enrichment in these metabolic processes was almost exclusively related to *ADH1A* and *ADH1B*.

**Table 5:** Gene Sets Analysis Results for Selected Gene Ontology (GO) Terms.

GO term	P (29 genes)	P (2 genes)
glycolysis/gluconeogenesis	1.45E-02	1.89E-03
fatty acid metabolism	2.52E-03	6.80E-04
retinol metabolism	2.66E-02	1.89E-03
ADH activity, Zn-dependent	2.86E-03	1.45E-05
ethanol metabolic process	8.28E-04	1.41E-05
ethanol oxidation	1.24E-02	1.33E-04

The final list of 29 most highly significant adipose genes was analyzed using Gene Profiler to detect enrichment of genes in various biological functional categories. A global set of all genes was used as the control group. P values are shown for several relevant GO categories containing *ADH1A* and *ADH1B*. Several categories contained only these two genes. The genes were analyzed as an ordered list based on FDR values and numbers of shared traits. Only manually curated data was used for the analysis to avoid potentially spurious results. Significance for these GO terms was increased approximately 10-fold when analysis was restricted to the two genes *ADH1A* and *ADH1B*. Essentially identical results were obtained using the WebGestalt analytical engine. All significance values were corrected for multiple testing.

### A Candidate Gene for Insulin Resistance/Obesity in Adipose Tissue

The expression of one gene, *ADH1A* (alcohol dehydrogenase 1A [class I] alpha polypeptide) (HGNC Symbol, Acc:249) was significantly correlated with BMI, WC and FPI. The expression of a second gene, *ADH1B* (alcohol dehydrogenase 1B [class I], beta polypeptide (HGNC Symbol, Acc:250) was significantly correlated with BMI and WC. In separate analyses these genes were not significantly, using the FDR cutoffs used here, correlated with BMI, WC, or FPI in skeletal muscle or leukocytes (data not shown). It is noteworthy that BMI and WC have the strongest phenotypic correlations among the examined traits in the entire VAGES dataset (**S2 Table**). *ADH1A* and *ADH1B* are highly homologous at the sequence and structural levels and are located on chromosome 4q23 within a cluster of 7 similar alcohol dehydrogenase genes (5 of which showed no significant correlation with the metabolic traits), all encoded in tandem on the reverse DNA strand. *ADH1A* is located approximately 1.5 Mb upstream (centromeric) of *ADH1B*. We used the Multi Experiment Matrix (MEM) tool from Gene Profiler to query *ADH1A*/*B* gene expression in 100 global gene expression datasets to search for significant gene expression patterns. The presence of both genes was highly significant across all datasets (4.47 x 10^–64^ and 6.31 x 10^–68^ for *ADH1A* and *ADH1B*, respectively). Interestingly, out of a total of 82 transcriptomic datasets analyzed, three of the most significantly similar datasets, with high expression of *ADH1A* and *ADH1B*, involved the study of adipocytes and adipocyte differentiation (“Programming human pluripotent stem cells into adipocytes” [E-GEOD-30038]; “Expression data of human perirenal adipose tissue-derived mesenchymal stem cells cultured under various conditions” [E-GEOD-18662]; and “Transcription profiling by array of human adipose-derived stem cells…”; [E-MEXP-3340]).

### High Expression of ADH1B compared with ADH1A in Adipose Tissue

According to the EMBL-EBI Expression Atlas (http://www-test.ebi.ac.uk) using data obtained by microarray assay of 76 normal human tissues (http://BioGPS.org) *ADH1B* is most highly expressed in adipose tissue, with almost double the level of expression found in the next highest tissue, liver (**S1 Fig.**). This finding was confirmed independently by high resolution sequencing of RNA (RNASeq) from 16 human tissues (Illumina Body Map 2.0). *ADHIB* had substantially higher expression in adipose tissue than in 15 other major tissues examined. Its expression was more than 3-fold higher in adipose tissue than in the next highest tissue, liver (**S2 Fig.**). In comparison, *ADH1A* expression by microarray was 208 fold lower than *ADH1B* in adipose tissue and the expression ratio in adipose vs. liver tissues was 1.87 for *ADH1B* compared with 0.0098 for *ADH1A*. The reason for the extremely high and specific expression of *ADH1B* in fat is currently unknown. Given these findings, we decided to examine the function of *ADH1B* in adipocytes in greater detail.

### Confirmative analysis of *ADH1B* using qRT-PCR

**Fig 2:**
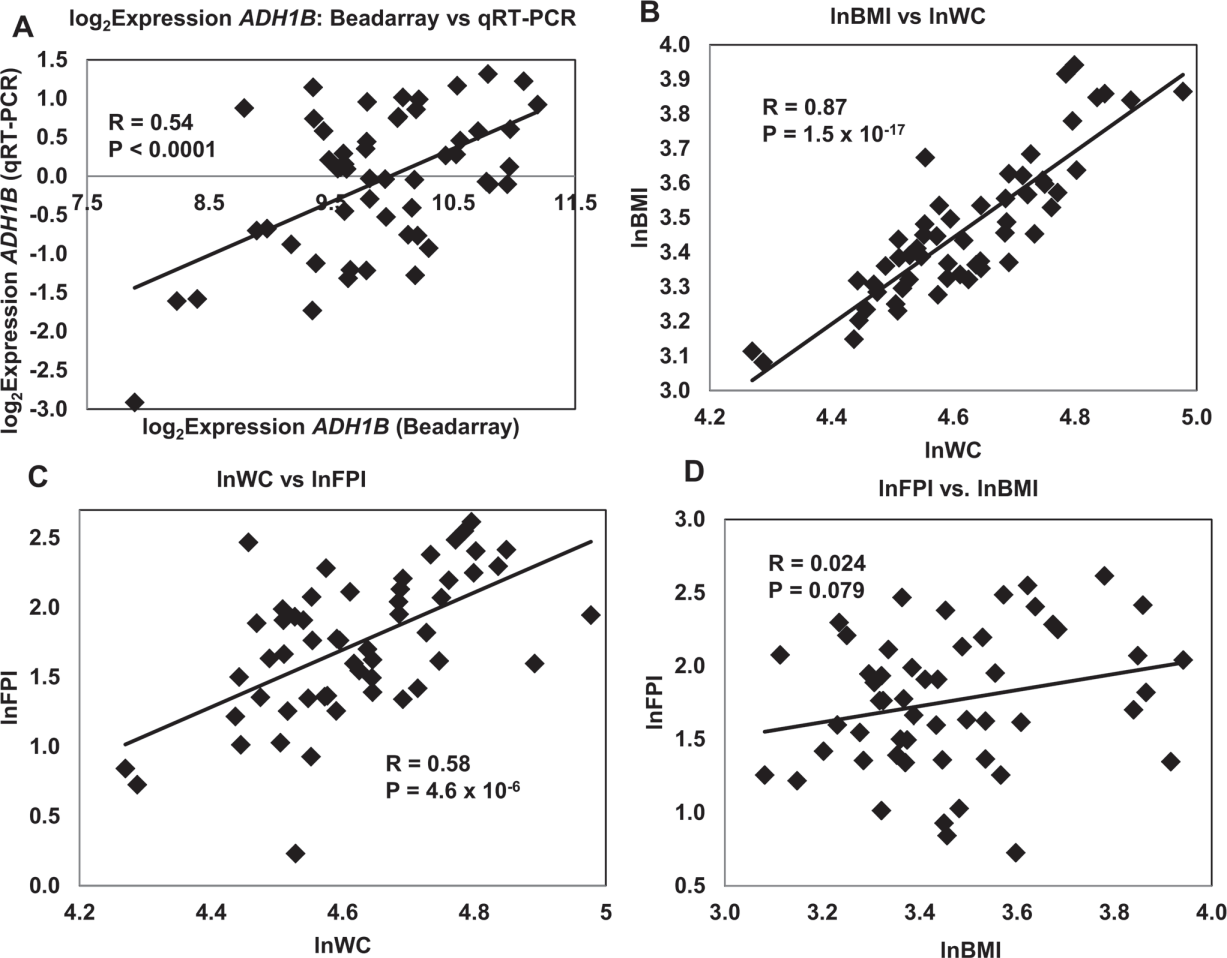
Comparisons of Gene Expression Methods (A) and Correlations between Three Key Obesity/Insulin Resistance Trait Pairs (B-D). Panel **A** shows the correlation of *ADH1B* expression measurements made separately with Illumina Beadarrays and qRT-PCR. Panels **B-D** show correlations between respectively, lnBMI vs. lnWC, lnWC vs. lnFPI, and lnFPI vs. lnBMI. Data were log transformed to minimize the issue of non-normality. qRT-PCR = quantitative real time polymerase chain reaction.

Despite the similarities between the *ADH1A* and *ADH1B* genes, our own data, and that from several other studies, taken together (see below), made a far stronger case for the putative involvement of *ADH1B*, rather than *ADH1A*, in adipose tissue metabolism associated with obesity and IR. Given this prior data we therefore focused most of our attention on the B isoform. We performed comparative gene expression measurements on the same fat RNA samples using an independent method, qRT-PCR, to precisely quantitate the transcript abundance of *ADH1B* present in fat samples. All qRT-PCR expression measures were converted to “Calibrated Normalized Relative Quantities” (CNRQs). We previously have shown good agreement between Affymetrix microarray expression data and qRT-PCR measurements of RNA expression (32). qRT-PCR was performed as previously described (32). Gene expression measured by Illumina BeadArrays was strongly correlated with qRT-PCR (R = 0.54, P = 2.9 x 10^–5^), (**Fig. 2 A**.) To assess the relationships between the three main variables associated with *ADH1B* we measured the correlations between the traits lnWC, lnBMI and lnFPI (**Fig. 2 B-D**). As expected, the strongest correlation was between lnWC and lnBMI (R = 0.87, P = 1.5 x 10^–17^) followed by lnWC and lnFPI (R = 0.58, P = 10^–6^). lnFPI and lnBMI were not significantly correlated. However, this may be due to the limited sample size since our prior bivariate analysis in non-diabetics from the entire VAGES cohort revealed a highly significant correlation between lnFPI and lnBMI including a strong genetic component (**S2 Table**). Taken together these data are consistent with a genetic mechanism underlying these traits, with possible pleiotropic influences.

**Fig 3:**
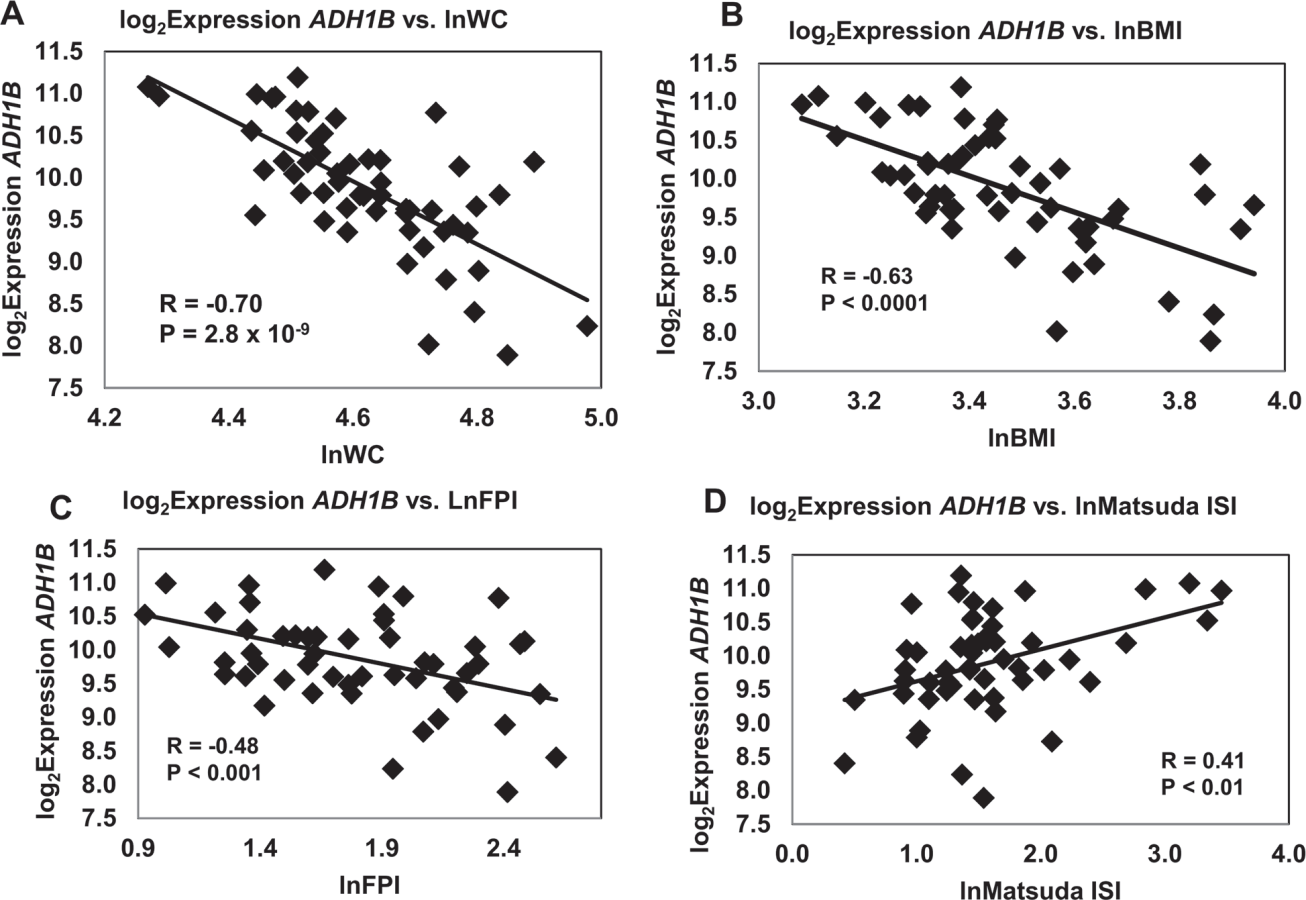
Correlation of *ADH1B* Expression with Key Traits (I). Log_2_ transformed gene expression measurements of *ADH1B* mRNA obtained by Illumina BeadArray were analyzed for correlation with **A** lnWC, **B** lnBMI, **C** lnFPI and **D** lnMatsuda Insulin Sensitivity Index. Variables were log transformed to minimize the issue of non-normality.

**Fig 4:**
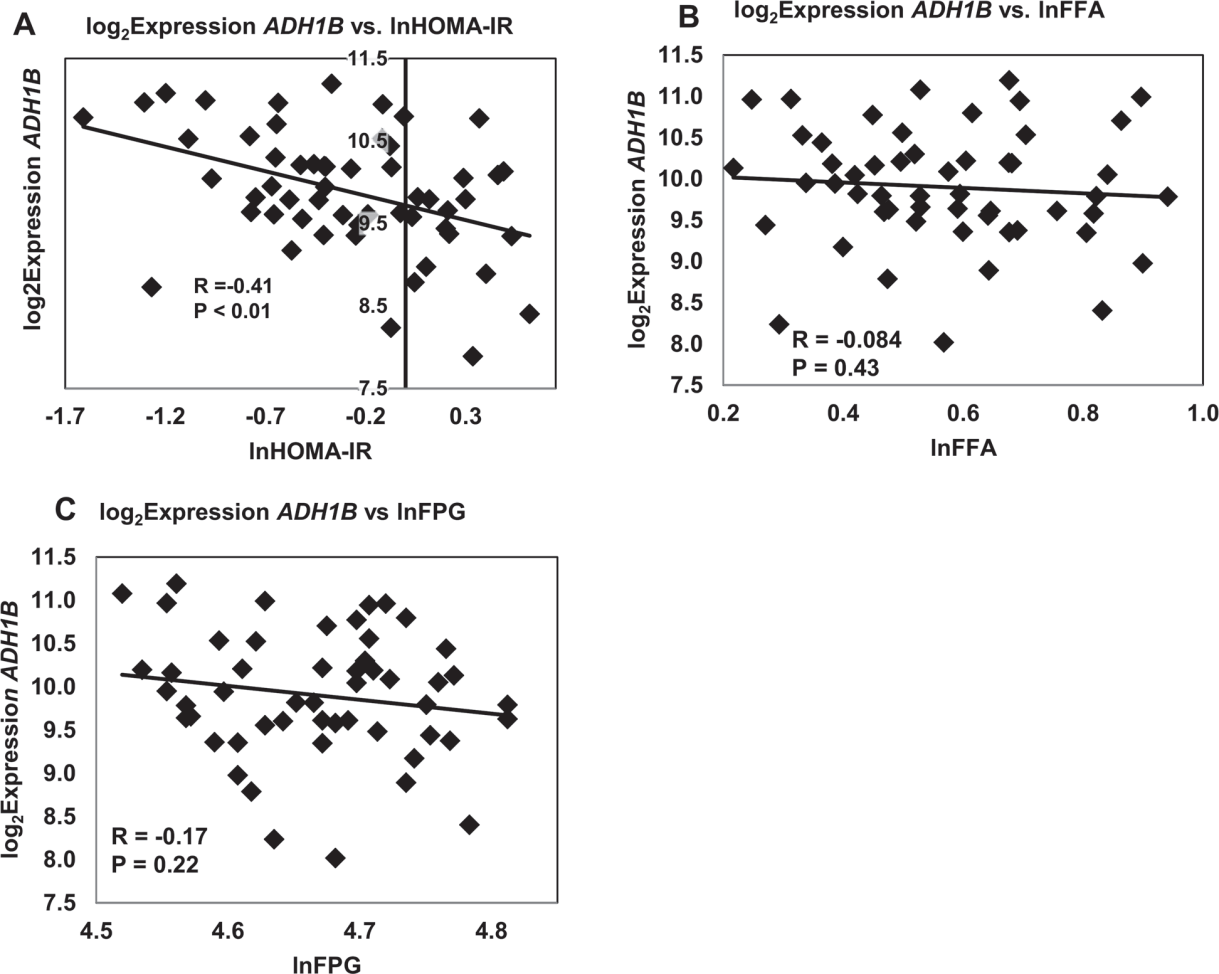
Correlation of *ADH1B* Expression with Key Traits (II). Log_2_ transformed gene expression measurements of *ADH1B* mRNA obtained by Illumina BeadArray were analyzed for correlation with **A** lnHOMA-IR, **B** lnFFA, **C** lnFPG. Variables were log transformed to minimize the issue of non-normality.

For illustrative purposes, we have provided a series of graphs showing the correlations between isolated *ADH1B* expression and key metabolic traits (Figs. 3–4). These results visually demonstrate the correlations which were used to initially identify *ADH1B* as a candidate gene strongly associated with obesity/IR traits. Variables were log transformed to minimize the issue of non-normality, and only batch 1 data, accounting for 80% of the fat samples, is shown (**Figs. 3 and 4**). Results using both Beadarray and qRT-PCR data, are summarized in **Table 6.** Correlations obtained using qRT-PCR were comparable and in agreement with those obtained with the Illumina BeadArray data. *ADH1B* gene expression in adipose tissue was strongly inversely correlated with lnWC (R = -0.70, P = 2.8 x 10^–9^), lnBMI (R = -0.63, P < 0.0001), lnFPI (R = -0.48, P < 0.001) and positively correlated with a measure of insulin sensitivity, the lnMatsuda Insulin Sensitivity Index (R = 0.41, P < 0.01) (**Fig. 3 A-D**). Consistent with these findings, *ADH1B* gene expression was inversely correlated with lnHOMA-IR (**Fig. 4 A**, R = -0.41, P < 0.01). No significant correlations were obtained for lnFFA and lnFPG (**Fig. 4 B-C)**.

**Table 6:** Summary of Results for Correlation of *ADH1B* Gene Expression with Key Traits.

Trait	Beadarray		qRT-PCR		N
	R	P	R	P	
lnWC	**-0.70**	**2.8 x 10** ^**–9**^	**-0.56**	**1.1 x 10** ^**–5**^	56
lnWC (NGT)	**-0.81**	**4.1 x 10** ^**–5**^	**-0.73**	**<0.001**	19*
lnWC (IFG)	-0.37	0.18	-0.36	0.19	15
lnWC (IGT)	-0.79	0.019	-0.55	0.16	8
lnWC (IFG/IGT)	**-0.86**	**8.6 x 10** ^**–5**^	**-0.64**	**<0.05**	14
lnBMI	**-0.63**	**5.4 x 10** ^**–6**^	**-0.58**	**5.0 x 10** ^**–5**^	56
lnBMI (NGT)	**-0.67**	**<0.01**	**-0.71**	**<0.001**	19*
lnBMI (IFG)	-0.48	0.067	-0.41	0.13	15
lnBMI (IGT)	-0.79	**<0.05**	-0.41	0.31	8
lnBMI (IFG/IGT)	**-0.75**	**<0.01**	**-0.67**	**<0.01**	14
lnFPI	**-0.48**	**<0.001**	**-0.34**	**<0.05**	56
lnFPI (NGT)	**-0.62**	**<0.01**	-0.58	0.014	19*
lnFPI (IFG)	-0.25	0.38	-0.20	0.50	15
lnFPI (IGT)	-0.43	0.29	-0.25	0.76	8
lnFPI (IFG/IGT)	**-0.74**	**<0.01**	-0.49	0.072	14
lnHOMA-IR	**-0.41**	**<0.01**	**-0.37**	**<0.01**	56
lnMatsuda ISI	**0.41**	**<0.01**	**0.37**	**<0.01**	56
lnFFA	-0.084	0.43	-0.02	0.87	56
lnFPG	-0.17	0.22	0.10	0.45	56

Correlation coefficients (R) and P values are shown for correlation analyses of *ADH1B* gene expression using both Illumina Beadarray signal intensity and qRT-PCR (calibrated normalized relative quantities) with key obesity and insulin resistance metabolic traits (lnWC, lnBMI, lnFPI, lnHOMA-IR, lnMatsuda ISI, lnFFA and lnFPG). Only FAT samples with both beadarray and qRT-PCR expression data in batch 1 were used in the analysis and the qRT-PCR correlations shown were not adjusted for sex and age. As shown, the three traits lnWC, lnBMI and lnFPI were also tested for correlation with IR/obesity traits after stratification of samples by pre-T2D status (i.e. NGT, IFG, IGT and IFG/IGT).

*One BeadArray sample was removed due to a low detection P value, as noted in the text.

### ADH1B Expression in Pre-Diabetes

To further elucidate the relationship of *ADH1B* with the key traits, lnWC, lnBMI and lnFFA we measured *ADH1B* expression in individuals stratified by pre-T2D status. We first stratified individuals by pre-T2D status (NGT, IFG, IGT and IFG/IGT). **S3**and **S4 Figs.** show FPG and FPI responses, respectively, during an OGTT in pre-diabetic individuals. **S5 Fig.** shows adipocyte IR in pre-T2D individuals. We measured *ADH1B* correlations within each pre-T2D group with each of the key traits (**S6–S8 Figs.**). Although the stratification diluted the number of individuals available for each analysis, clear trends emerged and are summarized in **Table 6**. The range of significant values was approximately 10^–2^–10^–5^ and the values were generally greater for the BeadArray data than the qRT-PCR data. Correlations were strongest for lnWC, followed by lnBMI and lnFPI. *ADH1B* expression was consistently associated, and at a similar level of significance within each key trait, with NGT and IFG/IGT. There was a modest trend towards significance for the IGT group but the sample size was relatively small (N = 8) compared with approximately twice that number in the other three pre-diabetes groups, NGT (N = 19), IFG (N = 15) and IFG/IGT (N = 14).

### ADH1B and Ethanol Consumption

Given the known enzymatic function of ADH1B in the oxidation of ethanol we examined the effect of drinking status on obese/IR correlations. Many epidemiological studies have reported little, no, or negative weight gain related to alcohol consumption although this is a multifactorial trait and can vary significantly within different cohorts. In our study cohort, 76% were moderate drinkers (National Institute on Alcohol Abuse and Alcoholism [NIAAA] definition) with an intake of 0.91 ± 1.07 (mean ± SD) standard drinks/day (NIAAA definition: 1 standard drink = 0.6 ounces of ethanol). We found a significant negative correlation between alcohol consumption [(drinks/d)^0.5^] and lnBMI (P<0.01, N = 55). We repeated correlations of *ADH1B* log_2_expression with lnBMI, lnWC and lnFPI, after adjusting for drinking level (plus age and sex covariates). Drinking was not independently significantly associated with these traits and when it was included in the model the significance of association was barely changed (WC: P = 1.2 x 10^–9^ changed to 1.8 x 10^–9^; BMI: 9.5 x 10^–9^ changed to 2.6 x 10^–9^; FPI: 2.0 x 10^–4^ changed to 1.3 x 10^–3^; N = 55). This indicates that the significant inverse correlations between *ADH1B* expression and obesity/IR traits were not confounded by drinking status.

### ADH1B Protein Expression is Inversely Correlated with BMI

**Fig 5:**
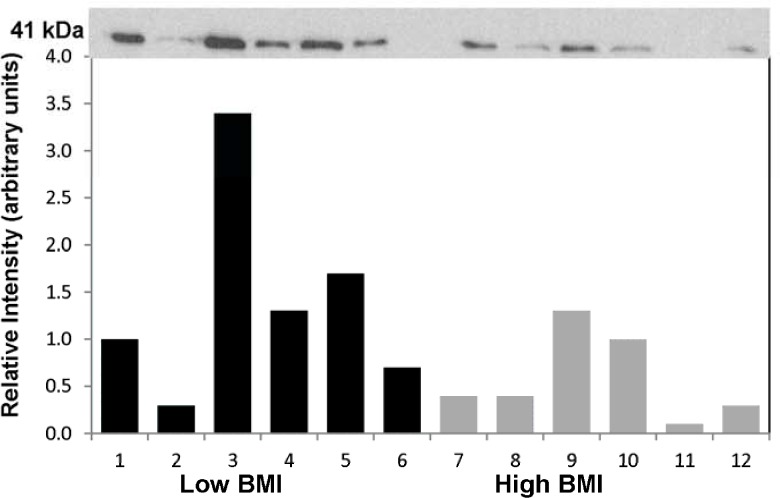
Immunoblot with Adipose Protein Samples from Subjects with High or Low BMI. Quantitation of adipose tissue protein obtained from biopsy lysates from subjects with high BMI (N = 6, BMI = 42.4 ± 7.3 kg/m^2^) and subjects with low BMI (N = 6, BMI = 27.5 ± 2.5 kg/m^2^) was performed by immunoblotting. Protein bands were corrected for relative loading using the Ponceau staining method. Lanes 1–6 BMIs: 22.5, 27.6, 28.0, 28.8, 28.9, 29.2. Lanes 7–12 BMIs: 34.3, 36.5, 36.9, 47.4, 47.7, 51.5. Mean relative signal ± SD: samples 1–6 = 0.37 ± 0.34, samples 7–12 = 0.08 ± 0.05 (P < 0.05, t-test, 1-tailed). Ratio of mean fluorescence intensity, low:high BMI = 4.6).

Given the strong correlative findings between *ADH1B* RNA expression and BMI we examined the relative levels of ADH1B protein in lysates obtained from adipose tissue biopsies from subjects with high BMI versus those with low BMI (**Fig. 5**). Quantitation of adipose tissue protein obtained from biopsy lysates from subjects with high BMI (N = 6, BMI = 42.4 ± 7.3 kg/m^2^) and subjects with relatively low BMI (N = 6, BMI = 27.5 ± 2.5 kg/m^2^) was performed by Western immunoblotting. Protein bands (at the expected size of 41 kDa) were clearly detected and corrected for relative loading using the Ponceau staining method. The mean (± SD) relative signals for the low BMI and high BMI groups were 0.37 ± 0.34, and 0.083 ± 0.055 (P < 0.05, t-test). The ratio of mean normalized fluorescence intensity, low:high BMI was 4.6. Thus the ratio of ADH1B protein expression in adipose tissue from the low BMI individuals was approximately 5-fold higher than that observed in the high BMI individuals, a finding consistent with the RNA correlation results.

## Discussion

Our overall goal was to discover the causal genes and associated variants which may underlie the genesis of T2D and related metabolic disorders. Although these studies are specific to the Mexican American population, which has a relatively high prevalence of T2D and related conditions, it is likely that similar gene functional effects will be generalizable to other populations manifesting the same disease-related traits.

*ADH1B* expression, measured both by Illumina BeadArrays and qRT-PCR, was negatively correlated with BMI, WC, FPI and HOMA-IR and positively correlated with the Matsuda ISI. It was not correlated with FPG or FFA. The lack of correlation with FFA could relate to the fact that about half of the FFA produced by lipolysis undergoes local reuptake and re-esterification by adipocytes and thus may not be an ideal measure of adipocyte function. These results clearly indicate decreased expression of *ADH1B* with higher levels of BMI, WC and FPI and hence an inverse relationship with obesity and IR. These relationships were also evident within subjects stratified by pre-T2D status. A negative correlation was found with WC, BMI and FPI in the NGT and IFG/IGT groups. The highly significant correlations of *ADH1B* expression with WC provide evidence for a potentially greater role for this gene in central obesity which is highly correlated with IR. It is not implausible that this relationship might be enhanced by the abdominal location of the adipose tissue biopsies, although they were subcutaneous rather than visceral. The RNA expression results were confirmed at the protein level by Western blotting. ADH1B protein expression was approximately 5-fold higher, on average, in adipose tissue from 6 low-BMI individuals compared with 6 high-BMI individuals. In summary, these direct correlations of *ADH1B* expression with various metabolic measures indicate a strong association of *ADH1B* with obesity and insulin resistance and a possible link with prediabetes.

The *ADH1A* and *ADH1B* genes are remarkably similar. They are located approximately 15 kb apart in a 380 kb Mb region on chromosome 4q23 that encodes a family of 7 homologous alcohol dehydrogenase genes in a tandem head-to-tail orientation. They share high structural and sequence homology at the DNA sequence level (>90% in exons, >70% in introns). Each has 9 exons and they appear to have arisen by gene duplication events, probably within the primates, with the divergence between *ADH1A* and *ADH1B* having occurred most recently.[37] The proteins share 94% amino acid sequence homology (sharing 355 of 375 aa) and have almost identical tertiary structures and enzymatic mechanisms.[44] Nevertheless despite this close similarity the expression of these two genes in adipose tissue is strikingly different.

Our selection of *ADH1B* rather than *ADH1A* was largely driven by the compelling evidence of substantially higher, and tissue-specific, expression of *ADH1B* in adipose tissues. Nevertheless, the fact that we have identified two very similar genes (in sequence, structure and enzymatic function) with highly significant correlation with multiple traits, provides stronger evidence since it argues for the relevance of the related functional pathway to the traits of interest. We searched the [38] for any *ADH1B*-related SNP with a threshold P value ≤ 10^–4^ (a P value of ≤ 10^–8^ is generally considered necessary to establish genome-wide significance). From a total of 682 studies with P values ≤ 10^–4^ only one SNP in a single study was significantly associated with *ADH1B* but the phenotype “upper aerodigestive tract cancers”[39] was clearly not related to the obesity/IR phenotypic association discovered in the present study. This underscores the relative difficulties of GWAS to be able to detect some types of genetic variation at the DNA level which may nevertheless be observable at the gene expression level, especially using precise, accurate physiological measures.

In a 2006 transcriptomic study of visceral adipose tissue gene expression in 5 type 2 diabetic vs 5 age- and BMI-matched normal glucose tolerant East Asian females, *ADH1B* showed significantly decreased expression in the diabetic patients (Gene Expression Omnibus [GEO] accession GSE16415; “Genome wide gene expression profiling of visceral adipose tissue among Asian Indian diabetics”). Gene expression was four-fold decreased in the diabetic patients vs the controls (52 vs 203 arbitrary units, respectively.) A transcriptomic study of obese vs non-obese subjects approximately equally divided into 3 T2D-related categories (T2D, IGT and NGT; total N = 33) found a significant decrease in *ADH1B* expression in each of the 3 obese groups compared with the matched controls (GEO accession GSE27951)[40].

ADH1B and other members of the ADH enzyme family metabolize a wide variety of substrates, including ethanol, retinol, other aliphatic alcohols, hydroxysteroids, and lipid peroxidation products. ADH1B can potentially enhance energy mobilization through two linked mechanisms. (1) It is involved directly in the reversible oxidation of ethanol to acetyl-CoA via acetaldehyde and acetate. Acetyl-CoA feeds directly into the Krebs cycle to be converted to CO_2_ and H_2_O with associated energy production. (2) It is involved in the reversible conversion of alcohol to fatty acids, which can then produce acetyl-CoA via β-oxidation. Therefore, elevated levels of ADH1B in human adipocytes could plausibly promote efficient metabolism of energy from alcohol, moderating its storage as fat. This would be consistent with our findings of higher *ADH1B* expression in adipose tissue of subjects with low BMI. (3) A third mechanism linking ADH1B with obesity/IR is its reversible conversion of retinal to retinol (vitamin A alcohol) from precursors. Retinol is specifically transported in the blood by the retinol binding protein (RBP4). RBP4 is an adipokine, secreted by adipocytes, that contributes to IR and obesity although there is some conflict between human and animal studies [41, 42]. Retinol and intracellular retinol binding proteins are also important for adipogenesis.

Apart from ethanol consumption, a second potentially important source of circulating ethanol, even in individuals who do not consume alcoholic beverages, comes from anaerobic fermentation of carbohydrates by microorganisms in the gastrointestinal tract (the microbiome)[43]. Humans are evolutionarily well adapted to utilize ethanol produced by microorganisms in the digestive tract[44] and perhaps elsewhere in the body.[45] Krebs demonstrated in 1970 that significant amounts of alcohol, at a rate of up to an ounce (30.4 g) of pure alcohol per day[46], are normally formed in the digestive tract. An important function of liver alcohol dehydrogenase (ADH, representing several similar enzymes) is the detoxification of normally occurring ethanol, with 75–90% being oxidized by the liver under normal conditions.[46] Numerous studies have provided extensive evidence for the presence of endogenous ethanol in the human circulatory system [46–54]. The levels of circulating endogenous ethanol are low compared with those from typical sources of exogenous ethanol. The lowest reported levels vary from 0.40 mg/l in normal healthy controls, to 6.6-fold higher levels in patients with T2D (2.64 mg/l, P < 0.01)[47], obesity[51] (2.6-fold) and in ob/ob genetically obese mice (3.5 fold).[55] Thus, endogenous ethanol represents a chronic background level of a readily metabolized fuel, second only to fat in caloric density. If 90% of this is oxidized by the Krebs cycle in the liver, the net ethanol in the bloodstream would be 3.04 g or 608 mg/l (in 5 l of blood). This is 1,520-fold higher than the normal mean circulating levels, indicating that approximately 3 g of ethanol may be oxidized elsewhere in extra-hepatic tissues. Thus, about 1% of the recommended daily food intake is available in the blood as endogenous ethanol for potential oxidation in extra-hepatic tissues. Under normal circumstances this additional source of energy is continually removed from the circulation and oxidized, maintaining a constant level of about 0.40 mg/l in the blood. However, if this regular energy supply is supplemented by higher daily food intake, augmented by ethanol intake, or enhanced by decreased energy expenditure, then the effect of this ethanol burning, or lack thereof, could be substantial. If the levels of the extra-hepatic ethanol burning enzyme(s) are relatively decreased then the effect will be even greater.

Ethanol that is not oxidized by liver ADH is available for metabolism elsewhere in the body. Adipose is one of the largest tissues in the body and one of the most complex and metabolically active, storing and furnishing energy under intricate hormonal control. Furthermore it has by far the highest expression of alcohol dehydrogenase 1B (*ADH1B*) RNA of all tissues examined. It is not currently known what contribution *ADH1B* activity in adipocytes may make to whole body ethanol clearance. However, as noted by Crabb and colleagues[56] ethanol metabolism has not been closely studied in obese individuals in whom adipose tissue might make a substantial contribution to alcohol elimination. For example, even if adipose tissue has 10% of the ADH activity of liver, obese individuals may have 50 times more adipose tissue than liver and adipose metabolism may make a substantial contribution to ethanol removal. This could vary depending on genetic and/or environmental factors. Thus, down-regulation of *ADH1B* expression in the adipose tissue of individuals who transition from lean to obese phenotypes could potentially exacerbate the worsening detrimental metabolic effects of increased adiposity. Many epidemiological studies have reported little, no, or negative weight gain related to alcohol consumption although this is a multifactorial trait and can vary significantly within different cohorts. In a population-based study of the relationship among alcohol intake, body fat, and physical activity, using data (N = 10,550 subjects) from the third National Health and Nutrition Examination Survey (NHANES), moderate and hazardous alcohol drinkers had significantly lower BMI (p < 0.01) and body weight (p < 0.01) than non-drinkers[57]. In the present study we also found a significant negative correlation between alcohol intake and BMI although alcohol consumption was not a significant covariate and did not affect the correlations between *ADH1B* expression and BMI, WC or FPI.

The 4q23 chromosomal region has previously been genetically linked to hypertension in a study in Mexican Americans living in San Antonio [58] and was recently linked to hip circumference in a study of T2D, obesity, dyslipidemia and hypertension in residents of the island of Kosrae, Federated States of Micronesia.[59] We previously found evidence for genetic linkage of small dense atherogenic LDL particles at chromosome 4q23 (LOD = 4.1) in Mexican Americans from the San Antonio Family Heart Study (SAFHS)[60]. In a study of adipogenic differentiation of mesenchymal stem cells (MSCs) from umbilical cord blood, genome-wide gene expression analysis identified significant (P = 3.03 x 10^–4^) upregulation of *ADH1B* during the change from undifferentiated to adipocyte-like differentiated MSCs.[61] The human chromosome 4q23 region has been shown to be homologous to a chromosomal region implicated in a murine model of abdominal obesity.[62] *ADH1B* is an adipogenic marker whose expression was limited by resveratrol-induced inhibition of clonal expansion and terminal differentiation of 3T3-L1 preadipocytes.[63]

It appears that the robust relationship observed between adipose tissue *ADH1B* gene expression with obesity/IR, at the RNA and protein level, may be present in both normal glucose tolerant and pre-diabetic individuals. It is, therefore, possible that the link between decreased *ADH1B* gene expression and obesity/IR which we have established in this study may exist both in individuals who do not ultimately develop T2D and those who are highly likely to develop this disease. However, this does not preclude a role for *ADH1B* in T2D given the fact that obesity and IR are primary risk factors for T2D. IR is present in approximately 25% of NGT individuals.[64] Given the multiple well established relationships between obesity/IR and severe metabolic disorders (including the metabolic syndrome, hypertension and cardiovascular disease) it remains the case that disordered regulation of *ADH1B* expression may be involved in manifold complex metabolic diseases with a high societal and economic burden. The functional nature of this relationship, and the specific role of *ADH1B* in adipose tissue, remains to be determined and warrants further investigation.

Taken together these findings provide strong experimental support for a potential role for adipose *ADH1B* in obesity/IR and T2D. This evidence includes high and specific expression in adipose tissue, strong inverse correlation with BMI, WC, FPI, HOMA-IR, pre-T2D and the Matsuda-ISI (positive correlation), highly significant association with glycolysis and fatty acid metabolism in gene sets analysis, highly significant expression across multiple transcriptomic studies, particularly those involving adipocytes and adipocyte differentiation, and supportive evidence from previous linkage studies, some of which were performed in the same target population of San Antonio Mexican Americans from which adipose tissue biopsies were donated for this study. In conclusion, we have established that there is a strong and consistent pattern of expression of human adipose *ADH1B* associated with various measures of obesity and IR. These findings are consistent with a central role for *ADH1B* in obesity and insulin resistance and provide evidence for a novel genetic regulatory mechanism for human metabolic diseases related to these traits.

## Materials and Methods

### Parent Study: The Veterans Administration Genetic Epidemiology Study (VAGES)

Demographic, genotypic and phenotypic data were collected from Mexican American individuals who were enrolled in the Veterans Administration Genetic Epidemiology Study (VAGES). VAGES families were ascertained on at least 2 siblings and one parent affected with T2D. The study procedures have been described elsewhere (24). The baseline clinical characteristics of all VAGES participants are reported in **S1 Table**. The VAGES baseline [65] plus its extension data analyses involved information from 1,546 individuals (females = 63.8%, mean age = 50.6 years, T2D = 58.4 and mean BMI = 37.3 kg/m^2^). The heritability (h^2^ ± SE) of T2D in VAGES data was determined to be 0.54 ± 0.11 after adjusting for the significant (*P* ≤ 0.05) covariate effects of age, sex, age^2^ and age^2^ x sex. The heritability estimates ranged from 0.18 (total cholesterol [TC]) to 0.59 (BMI), after adjusting for significant covariate effects of age and/or sex terms (S1 Table).

Phenotypic (ρ_P_), genetic (ρ_G_), and environmental (ρ_E_) correlations between selected T2D-related traits are shown in **S2 Table**. Correlations between T2D-related continuous traits were determined using bivariate genetic analysis and trait-specific covariates. The additive genetic correlation (ρ_G_) is a measure of the shared genetic basis of the two traits (i.e, pleiotropy). The environmental correlation (ρ_E_) indexes common nonshared environmental factors influencing the two traits. We partitioned the phenotypic correlations (ρ_P_) between the traits into genetic (ρ_G_) and environmental (ρ_E_) correlations. All ρPs between the trait pairs were highly significant, ranging from -0.32 (fasting insulin/non-diabetics and HDL cholesterol) to 0.73 (BMI and waist circumference [WC]). As expected, HDL-cholesterol was inversely correlated with fasting insulin, BMI, and fasting glucose. All ρGs were significantly influenced by common genetic factors (i.e., pleiotropy) excluding the trait pair fasting insulin and TG which was not significant. The significant ρGs ranged from -0.55 (fasting insulin/non-diabetics and HDL cholesterol) to 1.00 (fasting insulin/non-diabetics and SBP). As expected, HDL-cholesterol was inversely genetically correlated with fasting insulin, BMI, and fasting glucose. Only the ρEs between, fasting insulin and triglycerides (TG), fasting glucose and HDL-cholesterol, BMI and WC were statistically significant. In summary, these data reveal that phenotypic correlations among the trait pairs are largely influenced by genetic correlations and these trait pairs are under strong, significant common genetic or pleiotropic influences. These genetic influences underpin the gene expression effects on clinical traits examined in this study.

### Phenotypic Data

We used data from the VAGES to select 308 individuals for recall to undergo a standard OGTT with blood draws every 30 minutes. The phenotypic data for various metabolic and anthropometric traits, including medical history information, were collected for all study participants. FPG levels were measured during the OGTT. Subjects were classified as having T2D, if they had a fasting glucose concentration ≥ 126 mg/dl and/or 2h glucose level ≥ 200 mg/dl according to the 1999 criteria of the World Health Organization[66]. Participants who did not meet these criteria, but reported that they were under treatment with either oral anti-diabetic agents or insulin, and who gave a history of diabetes, also were considered to have T2D. Anthropometric data (i.e., height, weight, and WC) were collected using standardized procedures. BMI was calculated as weight in kilograms divided by the square of the height in meters (kg/m^2^). Based on glucose levels at zero and 120 minutes (t_0_, t_120_) of the OGTT, subjects were divided into four pre-diabetic categories: NGT, IFG, IGT, combined IFG/IGT, and T2D. Glycemic categories were defined as follows (glucose, mg/dl). (1) NGT: at t_0_ <100, and t_120_ <140; (2) IFG: at t_0_ ≥100 and <126 (3) IGT: at t_120_ ≥140 and <200; (4) IFG/IGT, as for IFG plus IGT; No subjects were T2D [67, 68]. The study protocol was approved by the Institutional Review Board of the University of Texas Health Science Center at San Antonio (UTHSCSA), and written informed consent was obtained from all subjects. All clinical procedures were performed at the Diabetes Clinical Center at the South Texas Veterans Health Care System Audie L. Murphy Hospital. Fasting blood samples were analyzed for relevant analyses including: FPG, glycated hemoglobin (HbA1c), FPI, TG, HDL, low density lipoprotein cholesterol (LDL), total cholesterol (TC) and free fatty acids (FFA). These were used to calculate well-validated whole body insulin resistance metrics including the homeostasis model assessment of IR HOMA-IR[34], the Matsuda Insulin Sensitivity Index[35] (measuring the reciprocal of IR), and adipocyte IR index (Adipo-IR).

### Pre-T2D and Adipose IR in the Study Subjects

Pre-diabetes (pre-T2D) categories were calculated from glucose levels before and at the end of the OGTT. These included impaired fasting glucose (IFG), impaired glucose tolerance (IGT), combined IFG/IGT in addition to normal glucose tolerance (NGT). We also measured relevant anthropometric variables including BMI, WC, systolic blood pressure (SBP) and diastolic blood pressure (DBP). Glucose, insulin and FFA were measured every 30 minutes during the OGTT in the 309 subjects. Subjects were partitioned by glycemic response into NGT, IFG, IGT, IFG/IGT or T2D. These categories relate to insulin sensitivity defects (i.e. IR) predominantly in liver (IFG), skeletal muscle (IGT) or both (IFG/IGT). There were severe IR defects in muscle and liver in IFG/IGT and T2D **(S1 Fig.)**. These IR differences also relate to whole body insulin response to the glucose challenge. In the more IR groups there was a diminished capacity for post-glucose insulin return towards basal levels **(S2 Fig.)** Decreased insulin action was also observed in adipose tissue. The more IR subjects also had deficient adipose insulin action, marked by elevated levels of FFA and an elevated adipocyte IR Index (i.e. lnFFA x FPI) **(S3 Fig.)**

### Processing of Gene Expression Data

The gene expression data were processed as follows: As a first step, we assessed the overall quality of the expression results of individual samples, based on the number of probes with significant detection (defined as having a “detection p-value” of ≤ 0.05), the average expression level (“mean raw expression level”) across all probes in a sample, and the average correlation across all probes of one sample versus all others (within a given tissue and batch). Only one sample (a fat sample) was found to be an outlier with poor quality, leaving 481 samples for further processing. Separately within each tissue and within batch, based on the “detection p-value” provided for each probe by Illumina software, we conducted a binomial test, assessing whether more samples of a given tissue in a given batch had a “detection p-value” ≤ 0.05 than expected by chance, before calculating the false discovery rate for a given probe in a given tissue and batch. Those probes significant at FDR 0.05 in any tissue batch (31545 probes, or 67% of all probes) were then subjected, within each tissue and batch, to background noise subtraction, log2 transformation, followed by quantile normalization within tissue and batch, another quantile normalization across tissues within a batch, and lastly a third quantile normalization across the three batches. All of the transcriptomic microarray data has been uploaded to the NCBI Gene Expression Omnibus data repository in MIAME-compliant format for public dissemination, accession ID number GSE64567.

### Statistical Analysis

A variance-components (VC) approach was used to estimate additive genetic influences on T2D and its related quantitative traits. In a simple model, variances or covariances between relatives as a function of the genetic relationships can be specified, and the proportion of phenotypic variance that is attributed to additive genetic effects (i.e., heritability: h^2^) can be estimated from the components of variance. The phenotypic, genetic, and environmental correlations between T2D-related traits were determined using bivariate genetic analysis. This is an extension of the VC approach where the phenotypic correlation (ρ_P_) between a pair of quantitative traits can be partitioned into additive genetic (ρ_G_) and environmental (ρ_E_) components. To address the issue of non-normality, for a given analysis, outliers, defined as ± 4 standard deviations from the mean were excluded. In addition, data were transformed using inverse normalization. For the inverse normalized data, which were used to determine additive genetic influences (S1 and S2 Tables), skewness values ranged from -0012 (diastolic blood pressure) to 0.0037 (BMI), and the kurtosis values ranged from -0.1596 (fasting insulin—non-diabetics) to -0.0813 (systolic blood pressure). In addition, we repeated all analyses using the log transformation. The findings were similar, and only the findings from the inverse normalized data were included in this report (S1 and S2 Tables). For the log transformed data, the skewness values ranged from -0.5946 (diastolic blood pressure) to 0.8665 (fasting glucose), and the kurtosis values ranged from -0.2127 (fasting glucose) to 0.6310 (total cholesterol). The metabolic trait data used for various sub-data set analyses were derived from these data sets. All genetic analyses, univariate (S1 Table) and bivariate (S2 Table), were performed using the VC approach as implemented in the computer program SOLAR [69]. All genetic analyses included adjustment for covariate effects of age and/or sex terms.

All gene, tissue and trait correlations were corrected for multiple testing using the False Discovery Rate [70]. Gene sets /pathway analyses were corrected using the default methods described by GeneProfiler [71] and WebGestalt [72]. Statistical approaches used to normalize transcriptomic data are described in the text. Briefly, the transcript values are fairly normal, due to multiple rounds of quantile normalization; and, the metabolic traits were log transformed to minimize the issue of non-normality.

Student’s t-test and ANOVA were used to evaluate trait differences between and among the diabetic and pre-diabetic groups (NGT, IGT, IFG, IGT/IFG, T2D).

## Supporting Information

S1 Fig RNA Expression of *ADH1B* in Human Tissues Measured by Microarray. *ADH1B* RNA expression was measured in a total of 76 normal human tissues and compartments hybridized against Affymetrix HG-U133A microarrays. Sixteen major tissues with the highest expression are shown. The Affymetrix MAS5 algorithm was used for array processing and probesets were averaged per gene. Data was downloaded from the BioGPS website (http://biogps.org) on December 24, 2013.
(TIF)

S2 Fig RNA Expression of *ADH1B* in Human Tissues Measured by RNASeq. *ADH1B* RNA expression was measured in a total of 16 normal human tissues by RNA sequencing and the sequences mapped to genes via their transcripts. The data was generated on HiSeq 2000 instruments in 2010. Expression is shown as Fragments Per Kilobase of exon per Million fragments mapped (FPKM) and was calculated using the Cufflinks program (http://cufflinks.cbcb.umd.edu/index.html). Illumina body map 2.0 data was downloaded from the Ensembl website (http://www.ensembl.info/) on December 24, 2013.
(TIF)

S3 Fig Plasma Glucose Response in Pre-Diabetics during the OGTT. Based on glucose levels at zero and 120 minutes (t_0_, t_120_) of the OGTT, 309 subjects were divided into NGT (normal glucose tolerant), IFG (impaired fasting glucose), IGT (impaired glucose tolerant), combined IFG/IGT, and T2D. Glycemic categories were defined as follows (glucose, mg/dl): (1) NGT at t_0_ <110, and t_120_ <140; (2) at t_0_ 110≥ IFG <126, and t_120_ <140; (3) at t_0_ <126, and t_120_ 140≥ IGT <200; (4) IFG/IGT, as for IFG plus IGT; (5) T2D, at t_0_ 126≥ T2D, and t_120_ 200≥ T2D. Complete data were available for: NGT (N = 55), IFG (N = 45), IGT (N = 24), IFG/IGT (N = 53) and T2D (N = 119). Values are mean ± SEM.
(TIF)

S4 Fig Plasma Insulin Response in Pre-Diabetics during the OGTT. Based on glucose levels at zero and 120 minutes (t_0_, t_120_) of the OGTT, 309 subjects were divided into NGT (normal glucose tolerant), IFG (impaired fasting glucose), IGT (impaired glucose tolerant), combined IFG/IGT, and T2D. Glycemic categories were defined as follows (glucose, mg/dl): (1) NGT at t_0_ <110, and t_120_ <140; (2) at t_0_ 110≥ IFG <126, and t_120_ <140; (3) at t_0_ <126, and t_120_ 140≥ IGT <200; (4) IFG/IGT, as for IFG plus IGT; (5) T2D, at t_0_ 126≥ T2D, and t_120_ 200≥ T2D. Complete data were available for: NGT (N = 55), IFG (N = 45), IGT (N = 24), IFG/IGT (N = 53) and T2D (N = 119). Values are mean ± SEM.
(TIF)

S5 Fig Adipocyte Insulin Resistance in Pre-Diabetics during the OGTT. Based on glucose levels at zero and 120 minutes (t_0_, t_120_) of the OGTT, 309 subjects were divided into NGT (normal glucose tolerant), IFG (impaired fasting glucose), IGT (impaired glucose tolerant), combined IFG/IGT, and T2D. Glycemic categories were defined as follows (glucose, mg/dl): (1) NGT at t_0_ <110, and t_120_ <140; (2) at t_0_ 110≥ IFG <126, and t_120_ <140; (3) at t_0_ <126, and t_120_ 140≥ IGT <200; (4) IFG/IGT, as for IFG plus IGT; (5) T2D, at t_0_ 126≥ T2D, and t_120_ 200≥ T2D. Complete data were available for: NGT (N = 55), IFG (N = 45), IGT (N = 24), IFG/IGT (N = 53) and T2D (N = 119). Values are mean ± SEM. Adipo-IR varied significantly among the 5 groups (*P* < 0.0001, ANOVA) and were increased in IFG, IFG/IGT and T2D compared with NGT (*p<0.001, **p<0.0001, t-test).
(TIF)

S6 Fig *ADH1B* Gene Expression Correlations with lnWC Stratified by Pre-Diabetes. Log_2_ transformed gene expression measurements of *ADH1B* mRNA obtained by Illumina BeadArray were analyzed for correlation with **A.** lnWC from NGT subjects, **B.** lnWC from IFG subjects, **C.** lnWC from IGT subjects and **D.** lnWC from IFG/IGT subjects.
(TIF)

S7 Fig *ADH1B* Gene Expression Correlations with lnBMI Stratified by Pre-Diabetes. Log_2_ transformed gene expression measurements of *ADH1B* mRNA obtained by Illumina BeadArray were analyzed for correlation with **A.** lnBMI from NGT subjects, **B.** lnBMI from IFG subjects, **C.** lnBMI from IGT subjects and **D.** lnBMI from IFG/IGT subjects.
(TIF)

S8 Fig *ADH1B* Gene Expression Correlations with lnFPI Stratified by Pre-Diabetes. Log_2_ transformed gene expression measurements of *ADH1B* mRNA obtained by Illumina BeadArray were analyzed for correlation with **A.** lnFPI from NGT subjects, **B.** lnFPI from IFG subjects, **C.** lnFPI from IGT subjects and **D.** lnFPI from IFG/IGT subjects.
(TIF)

S1 Table Characteristics of VAGES Participants and Heritability Estimates for Selected T2D-related Traits. The characteristics of the 1,546 VAGES participants are shown. Approximately 64% of the participants were female and the mean age was 50.6 years. The prevalence of T2D was 58.4% and the mean BMI was 37.3. The heritability (i.e., **h** ^**2**^) estimates ranged from 0.18 (total cholesterol) to 0.59 (BMI), after adjusting for significant covariate effects of age and/or sex terms.
(DOCX)

S2 Table Phenotypic (ρP), Genetic (ρG), and Environmental (ρE) Correlations between Selected T2D-Related Traits. After adjusting for trait-specific covariate effects, all ρPs between the trait pairs were highly significant, ranging from -0.32 (fasting insulin/non-diabetics and HDL cholesterol) to 0.73 (BMI and waist circumference [WC]). As expected, HDL-cholesterol was inversely correlated with fasting insulin, BMI, and fasting glucose. All ρGs were significantly influenced by common genetic factors (i.e., pleiotropy) excluding the trait pair fasting insulin and TG which was not significant. The significant ρGs ranged from -0.55 (fasting insulin/non-diabetics and HDL cholesterol) to 1.00 (fasting insulin/non-diabetics and SBP). As expected, HDL-cholesterol was inversely genetically correlated with fasting insulin, BMI, and fasting glucose. Only the ρEs between, fasting insulin and triglycerides (TG), fasting glucose and HDL-cholesterol, BMI and WC were statistically significant.
(DOCX)
