# Supplementary figures and images for: Transcriptomic Identification of ADH1B as a Novel Candidate Gene for Obesity and Insulin Resistance in Human Adipose Tissue in Mexican Americans from the Veterans Administration Genetic Epidemiology Study (VAGES)
Source: PLoS One. 2015 Apr 1;10(4):e0119941. doi: 10.1371/journal.pone.0119941 (PMC4382323; doi:10.1371/journal.pone.0119941)

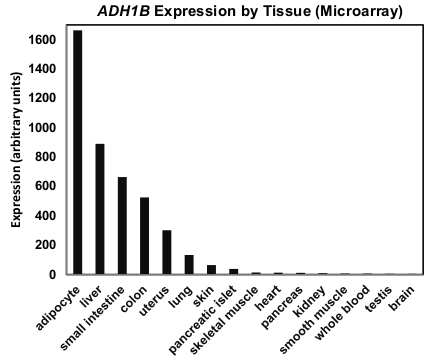

Supplement: S1 Fig — ADH1B RNA expression was measured in a total of 76 normal human tissues and compartments hybridized against Affymetrix HG-U133A microarrays. Sixteen major tissues with the highest expression are shown. The Affymetrix MAS5 algorithm was used for array processing and probesets were averaged per gene. Data was downloaded from the BioGPS website (http://biogps.org) on December 24, 2013. (TIF) [file pone.0119941.s001.tif]

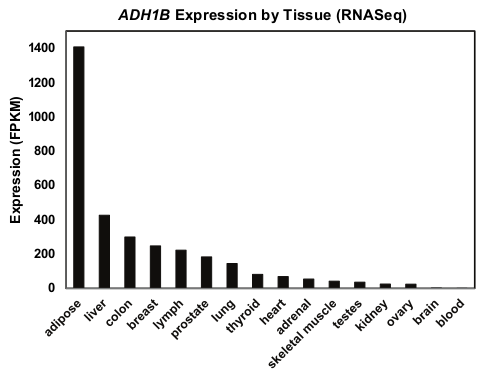

Supplement: S2 Fig — ADH1B RNA expression was measured in a total of 16 normal human tissues by RNA sequencing and the sequences mapped to genes via their transcripts. The data was generated on HiSeq 2000 instruments in 2010. Expression is shown as Fragments Per Kilobase of exon per Million fragments mapped (FPKM) and was calculated using the Cufflinks program (http://cufflinks.cbcb.umd.edu/index.html). Illumina body map 2.0 data was downloaded from the Ensembl website (http://www.ensembl.info/) on December 24, 2013. (TIF) [file pone.0119941.s002.tif]

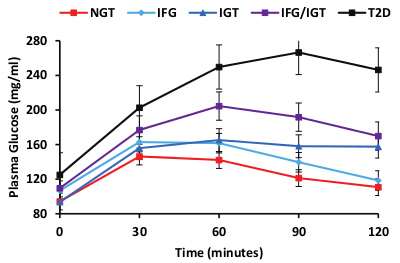

Supplement: S3 Fig — Based on glucose levels at zero and 120 minutes (t0, t120) of the OGTT, 309 subjects were divided into NGT (normal glucose tolerant), IFG (impaired fasting glucose), IGT (impaired glucose tolerant), combined IFG/IGT, and T2D. Glycemic categories were defined as follows (glucose, mg/dl): (1) NGT at t0 <110, and t120 <140; (2) at t0 110≥ IFG <126, and t120 <140; (3) at t0 <126, and t120 140≥ IGT <200; (4) IFG/IGT, as for IFG plus IGT; (5) T2D, at t0 126≥ T2D, and t120 200≥ T2D. Complete data were available for: NGT (N = 55), IFG (N = 45), IGT (N = 24), IFG/IGT (N = 53) and T2D (N = 119). Values are mean ± SEM. (TIF) [file pone.0119941.s003.tif]

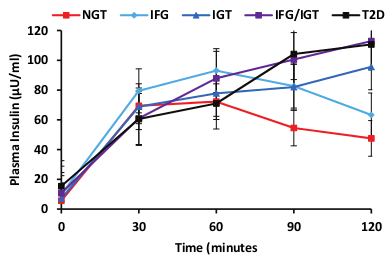

Supplement: S4 Fig — Based on glucose levels at zero and 120 minutes (t0, t120) of the OGTT, 309 subjects were divided into NGT (normal glucose tolerant), IFG (impaired fasting glucose), IGT (impaired glucose tolerant), combined IFG/IGT, and T2D. Glycemic categories were defined as follows (glucose, mg/dl): (1) NGT at t0 <110, and t120 <140; (2) at t0 110≥ IFG <126, and t120 <140; (3) at t0 <126, and t120 140≥ IGT <200; (4) IFG/IGT, as for IFG plus IGT; (5) T2D, at t0 126≥ T2D, and t120 200≥ T2D. Complete data were available for: NGT (N = 55), IFG (N = 45), IGT (N = 24), IFG/IGT (N = 53) and T2D (N = 119). Values are mean ± SEM. (TIF) [file pone.0119941.s004.tif]

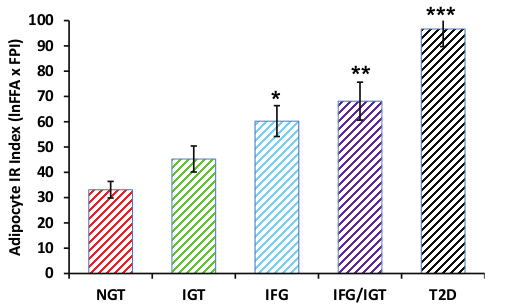

Supplement: S5 Fig — Based on glucose levels at zero and 120 minutes (t0, t120) of the OGTT, 309 subjects were divided into NGT (normal glucose tolerant), IFG (impaired fasting glucose), IGT (impaired glucose tolerant), combined IFG/IGT, and T2D. Glycemic categories were defined as follows (glucose, mg/dl): (1) NGT at t0 <110, and t120 <140; (2) at t0 110≥ IFG <126, and t120 <140; (3) at t0 <126, and t120 140≥ IGT <200; (4) IFG/IGT, as for IFG plus IGT; (5) T2D, at t0 126≥ T2D, and t120 200≥ T2D. Complete data were available for: NGT (N = 55), IFG (N = 45), IGT (N = 24), IFG/IGT (N = 53) and T2D (N = 119). Values are mean ± SEM. Adipo-IR varied significantly among the 5 groups (P < 0.0001, ANOVA) and were increased in IFG, IFG/IGT and T2D compared with NGT (*p<0.001, **p<0.0001, t-test). (TIF) [file pone.0119941.s005.tif]

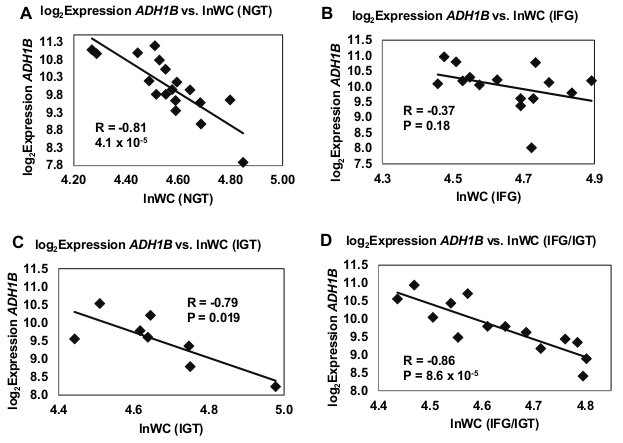

Supplement: S6 Fig — Log2 transformed gene expression measurements of ADH1B mRNA obtained by Illumina BeadArray were analyzed for correlation with A. lnWC from NGT subjects, B. lnWC from IFG subjects, C. lnWC from IGT subjects and D. lnWC from IFG/IGT subjects. (TIF) [file pone.0119941.s006.tif]

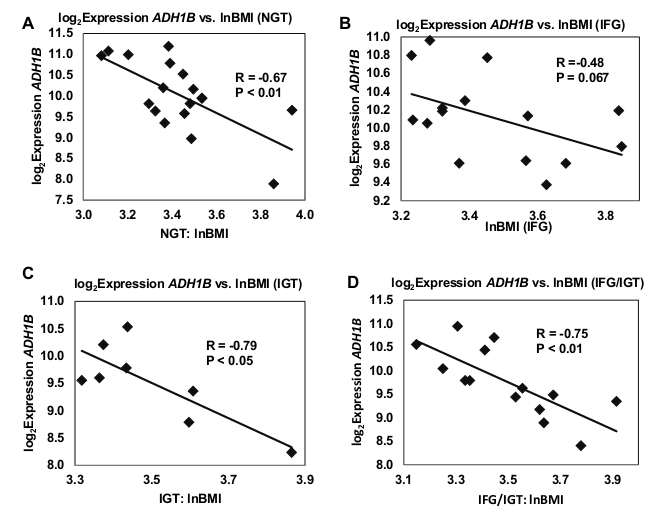

Supplement: S7 Fig — Log2 transformed gene expression measurements of ADH1B mRNA obtained by Illumina BeadArray were analyzed for correlation with A. lnBMI from NGT subjects, B. lnBMI from IFG subjects, C. lnBMI from IGT subjects and D. lnBMI from IFG/IGT subjects. (TIF) [file pone.0119941.s007.tif]

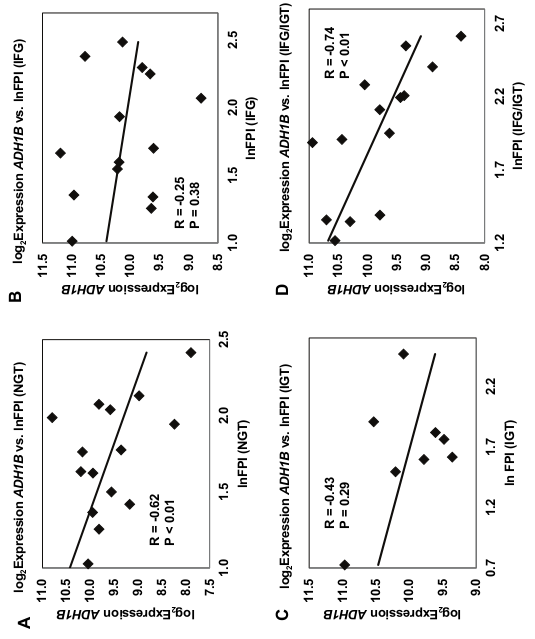

Supplement: S8 Fig — Log2 transformed gene expression measurements of ADH1B mRNA obtained by Illumina BeadArray were analyzed for correlation with A. lnFPI from NGT subjects, B. lnFPI from IFG subjects, C. lnFPI from IGT subjects and D. lnFPI from IFG/IGT subjects. (TIF) [file pone.0119941.s008.tif]
