# Supplementary material for: Transcriptomic Identification of ADH1B as a Novel Candidate Gene for Obesity and Insulin Resistance in Human Adipose Tissue in Mexican Americans from the Veterans Administration Genetic Epidemiology Study (VAGES)
Source: PLoS One. 2015 Apr 1;10(4):e0119941. doi: 10.1371/journal.pone.0119941 (PMC4382323; doi:10.1371/journal.pone.0119941)
Supplement: S1 Table — The characteristics of the 1,546 VAGES participants are shown. Approximately 64% of the participants were female and the mean age was 50.6 years. The prevalence of T2D was 58.4% and the mean BMI was 37.3. The heritability (i.e., h 2) estimates ranged from 0.18 (total cholesterol) to 0.59 (BMI), after adjusting for significant covariate effects of age and/or sex terms. (DOCX) [file pone.0119941.s009.docx]

| **Variable^a^** | **Mean ± SD or %** | **h^2^ ± SE** | ***P* value** | **Significant Covariates** |
| --- | --- | --- | --- | --- |
| **Females** | 63.8 |  |  |  |
| **Age (years)** | 50.6 ± 14.3 |  |  |  |
| **BMI (kg/m^2^)** | 37.3 ± 12.9 | 0.59 ± 0.06 | 2.7 x 10^-32^ | Sex, age |
| **Waist circumference (cm)** | 104.4 ± 17.6 | 0.42 ± 0.07 | 1.8 x 10^-14^ | Sex |
| **Type 2 Diabetes (T2D)** | 58.4 | 0.54 ± 0.11 | 3.0 × 10^-9^ | Age, sex, age^2^, age^2^×sex |
| **Fasting glucose (mg/dl)** | 141.7 ± 63.5 | 0.19 ± 0.05 | 5.4 x 10^-5^ | Age, sex, age^2^, age^2^×sex |
| **Fasting insulin[ND] (uU/ml )** | 14.7 ± 11.7 | 0.42 ± 0.17 | 0.0035 | - |
| **Total cholesterol (mg/dl)** | 183.5 ± 39.5 | 0.18 ± 0.06 | 7.7 x 10^-5^ | Age×sex, age^2^ |
| **HDL(mg/dl)** | 41.2 ± 11.9 | 0.48 ± 0.06 | 2.2 x 10^-21^ | Sex |
| **TG (mg/dl)** | 146.9 ± 93.7 | 0.37 ± 0.06 | 4.0 x 10^-13^ | Sex, age×sex |
| **SBP (mm Hg)** | 132.1± 19.4 | 0.25 ± 0.05 | 2.7 x 10^-8^ | Age, age×sex |
| **DBP (mm Hg)** | 74.1 ± 10.6 | 0.23 ± 0.06 | 3.4 x 10^-6^ | Sex, age^2^, age^2^×sex |
| ^a^ND = Non-diabetics | | | | |
