# Supplementary material for: Transcriptomic Identification of ADH1B as a Novel Candidate Gene for Obesity and Insulin Resistance in Human Adipose Tissue in Mexican Americans from the Veterans Administration Genetic Epidemiology Study (VAGES)
Source: PLoS One. 2015 Apr 1;10(4):e0119941. doi: 10.1371/journal.pone.0119941 (PMC4382323; doi:10.1371/journal.pone.0119941)
Supplement: S2 Table — After adjusting for trait-specific covariate effects, all ρPs between the trait pairs were highly significant, ranging from -0.32 (fasting insulin/non-diabetics and HDL cholesterol) to 0.73 (BMI and waist circumference [WC]). As expected, HDL-cholesterol was inversely correlated with fasting insulin, BMI, and fasting glucose. All ρGs were significantly influenced by common genetic factors (i.e., pleiotropy) excluding the trait pair fasting insulin and TG which was not significant. The significant ρGs ranged from -0.55 (fasting insulin/non-diabetics and HDL cholesterol) to 1.00 (fasting insulin/non-diabetics and SBP). As expected, HDL-cholesterol was inversely genetically correlated with fasting insulin, BMI, and fasting glucose. Only the ρEs between, fasting insulin and triglycerides (TG), fasting glucose and HDL-cholesterol, BMI and WC were statistically significant. (DOCX) [file pone.0119941.s010.docx]

| **Trait^a^** | **ρ_P_** | ***P* Value** | **ρ_G_ ± SE** | ***P* Value** | **ρ_E_ ± SE** | ***P* Value** |
| --- | --- | --- | --- | --- | --- | --- |
| **BMI-WC** | 0.73 | **3.1x10^-82^** | 0.83 ± 0.04 | **1.9x10^-18^** | 0.61 ± 0.05 | **7.2x10^-9^** |
| **FI/ND-BMI** | 0.47 | **1.5x10^-14^** | 0.59 ± 0.14 | **1.4x10^-3^** | 0.27 ± 0.29 | 0.3604 |
| **FI/ND-WC** | 0.46 | **4.0x10^-16^** | 0.53 ± 0.17 | **8.0x10^-3^** | 0.39 ± 0.20 | 0.1047 |
| **FI/ND-HDL** | -0.32 | **3.5x10^-8^** | -0.55 ± 0.18 | **3.8x10^-3^** | -0.15 ± 0.13 | 0.2896 |
| **FI/ND-TG** | 0.29 | **8.2x10^-7^** | 0.32 ± 0.19 | 0.1380 | 0.26 ± 0.12 | **5.0x10^-2^** |
| **FI/ND-SBP** | 0.19 | **6.3x10^-4^** | 1.00^b^ | **3.9x10^-2^** | -0.02 ± 0.14 | 0.8619 |
| **BMI-HDL** | -0.24 | **4.6x10^-7^** | -0.40 ± 0.13 | **7.1x10^-3^** | 0.06 ± 0.24 | 0.7958 |
| **FG-HDL** | -0.26 | **3.4x10^-13^** | -0.39 ± 0.15 | **1.6x10^-2^** | -0.22 ± 0.07 | **1.3x10^-3^** |

^a^FI = fasting insulin/ND = nondiabetic individuals only; ^b^went to bound.
